# Long-term nitrogen management strategies based on straw return improve soil carbon and nitrogen fractions and nitrogen use efficiency of maize in the Tumochuan Plain Irrigation District

**DOI:** 10.3389/fpls.2025.1620311

**Published:** 2025-08-21

**Authors:** Wenbo Li, Jiawei Qu, Julin Gao, Xiaofang Yu, Daling Ma, Shuping Hu, Qinggeer Borjigin, Kexin Lu

**Affiliations:** ^1^ College of Agriculture, Inner Mongolia Agricultural University, Hohhot, China; ^2^ Key Laboratory of Crop Cultivation and Genetic Improvement of Inner Mongolia Autonomous Region, Hohhot, China; ^3^ Vocational and Technical College, Inner Mongolia Agricultural University, Baotou, China; ^4^ Institute of Biotechnology, Inner Mongolia Tongliao Agricultural and Animal Husbandry Academy, Tongliao, China

**Keywords:** straw return, nitrogen use efficiency, soil fertility, maize yield, sustainable agriculture

## Abstract

**Introduction:**

Straw return combined with rational nitrogen (N) fertilization plays a critical role in coordinating the transformation of soil organic carbon and nitrogen availability, thereby improving nitrogen use efficiency (NUE), crop yield, and soil fertility. However, the dynamics of soil carbon and nitrogen fractions under straw return with varying N inputs, and their specific contributions to NUE and yield, remain unclear.

**Methods:**

A three-year split-plot field experiment was conducted in the Tumochuan Plain Irrigation District. The main plots included deep plowing with straw return (DPR) and no straw return (RT), while subplots comprised four N application rates (0, 210, 255, and 300 kg ha^-1^). Soil carbon and nitrogen fractions, maize yield, NUE, and partial factor productivity of nitrogen (PFPN) were assessed.

**Results:**

Compared to RT, DPR significantly improved soil nutrient levels and labile C and N fractions in the 0–40 cm soil layer. Maize yield, NUE, and PFPN increased by 17.28%, 18.24%, and 17.88%, respectively. Under DPR, a linear-plus-plateau model estimated the optimal N rate at 237.3 kg ha^-1^, reducing N input by 20.89% without compromising performance. Key contributors to NUE and PFPN included mineral nitrogen (MN), soil quality index (SQI), and dry matter accumulation (DMA), with relative contributions of 9.39%, 8.96%, and 8.49% to NUE, and 9.31%, 9.18%, and 8.99% to PFPN, respectively.

**Discussion:**

Straw return enhanced soil nitrogen availability and maize productivity by improving MN and SQI. Even with a 15–20% reduction in N application, DPR sustained high soil C and N fractions, yield, and NUE. These results offer practical guidance for optimizing N management under long-term straw return, with significant implications for sustainable maize production and soil fertility enhancement.

## 1 Introduction

With advancements in agricultural technology, continuous increases in crop yields have led to a dramatic rise in crop straw production (Ren et al., 2019). However, traditional farming practices, such as straw removal and excessive nitrogen fertilization, have resulted in severe soil fertility degradation and unsustainable dependence on external nitrogen inputs (Hou et al., 2020). As a simple yet effective method for managing crop by products, straw return not only helps maintain soil organic matter and enhance biological activity, but also improves soil physical properties and increases nutrient availability (Chen et al., 2014a). Long-term sole nitrogen fertilization leads to low nitrogen use efficiency (NUE) and diminishing yield-increasing effects, while excessive nitrogen application can cause environmental pollution (Wang et al., 2020). Compared to straw application alone, the combined application of straw and chemical fertilizers significantly increased soil labile organic carbon content and enhanced microbial functionality (Liu et al., 2022), However, excessive nitrogen fertilizer application has conversely generated negative environmental impacts (Ma et al., 2022), which underscores the urgency of rationalizing nitrogen management. To mitigate the environmental pressure, nitrogen fertilizer application rates are typically strictly controlled. Therefore, appropriate quantification of straw return and nitrogen fertilizer input under different cultivation conditions and research objectives is critical for regulating the relationship between soil organic carbon accumulation and nitrogen availability, thereby achieving sustainable crop yield, enhanced NUE, and improved soil fertility.

Soil carbon sequestration is a key strategy for mitigating climate change and enhancing soil fertility (Wiesmeier et al., 2014), straw return is a vital practice for enhancing and maintaining soil organic carbon (SOC). Research demonstrates that 15-year continuous straw return tripled cumulative carbon input while increasing SOC content by 14.2% compared to initial soil levels (Hao et al., 2022). As a major source of SOC in agricultural systems, straw return facilitates soil particle aggregation and promotes the formation of soil aggregates, consequently modifying SOC distribution among different functional fractions (Huang et al., 2018). Based on SOC turnover rates, SOC can be categorized into labile SOC fractions (e.g., MBC, microbial biomass carbon; EOC, easily oxidizable carbon; DOC, dissolved organic carbon) and stable SOC fractions (e.g., MAOC, mineral-associated organic carbon). The increase in the rate of straw returning to the field has significantly enhanced the turnover rate of soil organic carbon. while nitrogen fertilization further amplified this effect under straw-returned conditions. Notably, the interaction between straw return and nitrogen application markedly increased both the content and proportion of labile organic carbon in soils (Li et al., 2019b; Jha et al., 2020). However, despite these findings, the impacts of combined straw return and nitrogen input on SOC fractions, especially in field conditions involving tillage, remain insufficiently understood.

The biogeochemical cycles of soil carbon and nitrogen are closely coupled, and nitrogen dynamics are substantially influenced by straw return. The combined application of straw return and nitrogen fertilizer alters soil structure, moisture content, and the C/N ratio, thereby enhancing microbial activity and promoting nitrogen fixation (Li et al., 2019a). Straw return not only contributes additional nitrogen inputs, but also enhances nitrogen retention, reduces nitrogen leaching, and promotes nitrogen accumulation in the soil (Xia et al., 2018). Various complex nitrogen transformation processes, including mineralization, nitrification, and denitrification, occur simultaneously in the soil. Variations in the rates of these processes play a critical role in crop nitrogen uptake and nitrogen loss pathways. Moreover, long-term straw return combined with nitrogen fertilization significantly enhances the primary mineralization rate of soil nitrogen (Yu et al., 2024). The interactions between carbon and nitrogen components are crucial for understanding nutrient cycling and improving crop productivity (Kulagowski et al., 2021). However, most existing studies have not clearly disentangled the effects of reduced nitrogen input under long-term straw return systems. In particular, little is known about the contribution of specific carbon and nitrogen fractions to maize productivity and nitrogen use efficiency in systems involving deep plowing with straw return (Zhang et al., 2024b).

This research explores the combined impact of straw return and nitrogen reduction, aiming to elucidate the potential benefits of this practice for soil fertility and crop productivity. The study also seeks to understand how soil fertility and labile carbon and nitrogen fractions influence maize yield and nitrogen use efficiency (NUE), and how nitrogen application can be optimized under long-term straw return conditions to sustain crop productivity and soil health. Given the growing emphasis on sustainable agricultural practices, this study seeks to clarify the contributions of soil fertility and labile carbon and nitrogen fractions to crop yield and nitrogen efficiency, and to determine the optimal nitrogen application rate under multi-year straw return conditions, providing theoretical support for nitrogen management strategies in straw return systems.

## 2 Materials and methods

### 2.1 Description of research location

**Figure 1:**
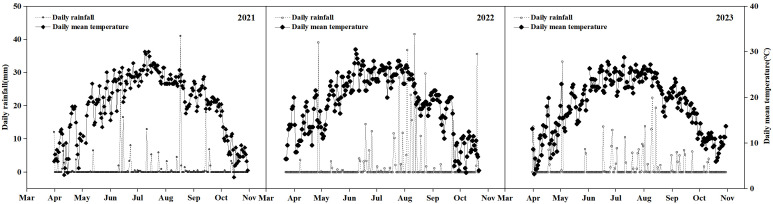
Main meteorological factors during the growing period in the experimental area.

Field experiments were conducted from 2021 to 2023 at the China Chilechuan Modern Agricultural Expo Park in Tumoteyou Qi, Baotou City, Inner Mongolia Autonomous Region, China. The region is located in the Tumochuan Plain, which has a temperate continental monsoon climate. Its geographical coordinates are 40°28′28″N latitude and 110°29′5″E longitude. The soil type is sandy loam. The baseline physicochemical properties of the 0–40 cm soil layer under different tillage treatments prior to the experiment are presented in **Table 1**, while the main meteorological factors during the experimental period are shown in **Figure 1**. The average temperatures during the growing seasons of 2021, 2022, and 2023 were 18.4°C, 18.6°C, and 18.3°C, respectively, and the corresponding average precipitation values were 205.9 mm, 413.92 mm, and 294.6 mm.

**Table 1:** Base productivity of the test site.

Tillage	Soil organic carbon (g kg^-1^)	Available P (mg kg^-1^)	Available K (mg kg^-1^)	Alkali-hydrolyzable Nitrogen (mg kg^-1^)	Total nitrogen (g kg^-1^)	Bulk density (g cm^-3^)	pH
RT	12.39	12.66	80.52	59.68	1.45	1.57	7.56
DPR	13.24	15.19	94.73	64.80	1.51	1.5	7.18

### 2.2 Experimental materials

The spring maize variety used in the experiment was XY335.

### 2.3 Experimental design

Before the experiment, the planting method of the plots was single spring maize (Zea mays L.) continuous crop-ping. The experiment was conducted using a split-plot design with two factors: straw return treatments and nitrogen application rate. The main plots were assigned to straw return treatments, including deep plowing with straw return (DPR) and no straw return (RT). The sub-plots included four nitrogen application rates: normal application (N3, 300 kg ha^-1^), 15% reduction (N2, 255 kg ha^-1^), 30% reduction (N1, 210 kg ha^-1^), and no nitrogen application (N0, 0 kg ha^-1^). In the deep plowing with straw return treatment, maize straw was first mechanically crushed after harvest and then incorporated into the soil through deep plowing to a depth of 35–40 cm. In the treatment without straw return, the crushed straw was removed from the field using mechanical extraction. The quantities of straw returned under deep plowing were 26,504.78 kg ha^-1^ in 2021, 27,532.32 kg ha^-1^ in 2022, and 29,145.19 kg ha^-1^ in 2023. Nitrogen was applied as urea (46% Nitrogen) in a 3:7 split between the jointing stage and the large trumpet stage. Phosphorus and potassium fertilizers were applied as basal fertilizers before sowing, including calcium superphosphate (12% P_2_O_5_, 105 kg ha^-1^) and potassium sulfate (60% K_2_O, 45 kg ha^-1^). The area of each experimental plot is 180 m^2^ (30 m×6 m). The planting density was 82,500 plants ha^-1^, and each treatment was replicated three times. A total of seven irrigations were applied during the growing season, specifically before sowing, at the jointing stage, large trumpet stage, silking stage, and 15, 30, and 45 days after silking, with an irrigation amount of 515 m³ ha^-1^ each time. All other field management practices followed conventional practices for large-scale maize cultivation.

### 2.4 Calculation of soil available nutrients and soil quality index

Prior to maize sowing each year, soil samples from the 0–40 cm tillage layer were collected using the five-point sampling method. The samples were thoroughly homogenized, placed in sealed bags, and transported to the laboratory. After air-drying in a shaded area, visible roots and other debris were removed. The soil was then ground and passed through a 1 mm sieve for the determination of soil organic carbon (SOC), total nitrogen (TN), alkali-hydrolyzable nitrogen (AN), available phosphorus (AP), and available potassium (AK).

SOC was determined using the potassium dichromate external heating method (Walkley and Black, 1934). TN was measured by the semi-micro Kjeldahl method (Bremner, 1960), and AN was analyzed using the alkali diffusion method (Norman, 1965). AP was extracted with sodium bicarbonate and quantified by the molybdenum-antimony anti-colorimetric method (Olsen et al., 1954), while AK was determined by flame photometry (Murphy and Riley, 1962).

Calculation of SQI: The SQI was evaluated using the total dataset method (Nabiollahi et al., 2018). First, all soil available nutrient (SOC,TN,AN,AP,AK) values were transformed into dimensionless scores ranging from 0 to 1. A linear scoring model was employed, as described below:

Increasing Membership Function:


$$XL=\{\begin{array}{ll} 0.1 & \text{X}\leq \text{X}1\\ 0.9(\text{X}-\text{X}1)/(\text{X}2-\text{X}1)+0.1 & \text{X}1<\text{X}\\ 1.0 & \text{X}\geq \text{X}2 \end{array}\}$$

Decreasing Membership Function:


$$XL=\{\begin{array}{ll} 0.1 & \text{X}>\text{X}2\\ 0.9(\text{X}2-\text{X})/(\text{X}2-\text{X}1)+0.1 & \text{X}1<\text{X}\\ 1.0 & \text{X}\leq \text{X}2 \end{array}\}$$

where *XL* represents the linear score (ranging from 0 to 1), *x* is the measured value of the indicator, *x_2_* and *x_1​_* are the minimum and maximum values of the indicator, respectively. Subsequently, the weights (*Wi*) of each indicator were determined through principal component analysis (PCA), calculated as the ratio of their variance to the cumulative variance. The weights of soil indicators were determined using principal component analysis (PCA). After standardizing all indicators (SOC, TN, AN, AP, AK), components with eigenvalues >1 were retained. For each indicator, its absolute loading values across the selected components were multiplied by the respective explained variance. The final weight of each indicator was calculated as the proportion of its weighted contribution to the total. These weights were then applied in the weighted summation for SQI calculation. The SQI was then computed using the following formula:


$$SQI=\sum_{i=1}^{n}wi\times SL$$

where *Wi* is the weight of the *i*-th evaluation indicator, *SL* is the score of the indicator, and *n* is the number of indicators.

### 2.5 Determination of soil labile carbon and nitrogen fractions

During the silking stage of maize each year, soil samples from the 0–40 cm tillage layers were collected using the five-point sampling method. The samples were thoroughly homogenized, placed in sealed bags, and transported to the laboratory. A portion of each sample was stored at −20°C for the determination of dissolved organic carbon (DOC), microbial biomass carbon (MBC), and microbial biomass nitrogen (MBN). The remaining portion was air-dried in a shaded area, with visible roots and other debris removed. The dried soil was then ground, passed through a 1 mm sieve, and used for the determination of easily oxidizable organic carbon (EOC). Carbon and nitrogen fractions in this study were classified based on their lability and microbial availability. Specifically, EOC, DOC, and MBC represent labile and microbially active carbon pools, while MBN and MN indicate available and mineral nitrogen pools involved in short-term nutrient cycling.

DOC was extracted using ultrapure water at a 1:10 (w/v) soil-to-water ratio and quantified using a TOC analyzer (Chen et al., 2019). EOC was determined by potassium permanganate oxidation (Jiang et al., 2021). MBC and MBN were determined by chloroform fumigation-K_2_SO_4_ leaching (Vance et al., 1987).

During the silking stage each year, soil samples were collected from the 0–100 cm soil profile using the five-point sampling method. The samples were divided into five depth intervals: 0–20 cm, 20–40 cm, 40–60 cm, 60–80 cm, and 80–100 cm. After thorough homogenization, the samples were placed in sealed bags and transported to the laboratory. All soil samples were stored at −20°C for the determination of mineral nitrogen (MN) concentrations.

Soil MN content was the sum of soil NO_3_^–^N and NH_4_^+^-N, which were extracted using 2 mol L^−1^ KCL, Subsequently, measurements were performed using a Smartchem 450 fully automatic chemical analyzer.

### 2.6 Plant dry matter accumulation and nitrogen uptake by plants

At maize maturity each year, three uniformly growing plants were selected from each plot. Aboveground parts, including stems, leaves, and ears, were separated and placed into mesh bags. The samples were initially heated at 105°C for 30 minutes to deactivate enzymes, then oven-dried at 80°C to a constant weight. After drying, each organ was weighed using a 0.01 g analytical balance to calculate aboveground DMA. The dried samples were then ground and passed through a sieve, and the total nitrogen content was determined using the Kjeldahl method (Bremner, 1960). NUP was calculated accordingly.

### 2.7 Yield and nitrogen efficiency calculation

Plot yield measurement was conducted at harvest. Two inner rows per treatment were selected, each with a length of 5 meters, and the actual harvested area was calculated (The actual harvested area was 6m^-2^). Within this area, the total number of plants, number of ears, number of double ears, number of barren stalks, number of lodged plants, number of plants actually harvested, and total number of ears harvested were recorded. Yield was calculated based on the actual harvested area.

Nitrogen Fertilizer Use Efficiency Calculation Formula:

The NUE was used to characterize the absorption and utilization efficiency of crops for applied nitrogen fertilizer. NUE was calculated as follows:


$$\begin{array}{c} \text{Nitrogen Use Efficiency}(\text{NUE},\%)=\\ \frac{\left(\text{N uptake by plants in the nitrogen application area}-\text{N uptake by plants without nitrogen application}\right)}{\text{N application}}*100\% \end{array}$$

(Dobermann and Cassman, 2005).

The PFPN was used to describe the use efficiency of fertilizer nitrogen for maize yield, PFPN was calculated as follows:


$$\text{Partial Factor Productivity of Nitrogen}(\text{PFPN},\;{\text{kg kg}}^{-1})=\frac{\text{Grain yield}}{\text{Nitrogen application}}$$

(Ladha et al., 2005)

### 2.8 Statistical analysis

Microsoft Excel 2019 (Microsoft, Inc., Redmond, WA, USA) was used to organize and analyze the data, and SAS 9.4 (SAS Institute Inc., Raleigh, NC, USA) statistical software was employed for split-plot analysis of variance (ANOVA). Multifactorial ANOVA was conducted using the LSD (least significant difference) method, with a significance level of *p* < 0.05, and multiple comparisons were performed using Duncan’s test. The interrelationships among soil carbon and nitrogen, plant DMA, yield, and nitrogen efficiency were examined using Origin 2021 (OriginLab Corp., Northampton, MA, USA) to generate linear-plus-plateau prediction models and principal component analysis (PCA) plots. R statistical programming language was applied to establish a random forest model and perform correlation analysis.

## 3 Results

### 3.1 Soil available nutrients

As shown in **Table 2**, year, straw return, and nitrogen application rate all had highly significant effects on the contents of soil alkali-hydrolyzable nitrogen (AN), available phosphorus (AP), and available potassium (AK). However, under the three-way interaction of these factors, only the AN content was significantly affected. Compared with the RT treatment, the DPR treatment significantly increased AN, AP and AK contents (*p*<0.05), with increases over the three years ranging from 10.25% to 24.83%, 11.20% to 25.52% and from 17.64% to 22.98%, respectively. After three years of straw return, the AN, AP, and AK contents increased by 21.37%, 13.03%, and 5.07%, respectively. Under the DPR treatment, these nutrient levels decreased with reduced nitrogen application. Compared with N3, the N1 treatment significantly reduced AN, AP, and AK by 12.17%, 11.71%, and 6.69% (*p*<0.05), respectively. Although these nutrient levels were slightly lower in the N2 treatment than in N3, the differences were not statistically significant (*p*<0.05).

**Table 2:** Soil available nutrient contents under different straw return treatments combined with nitrogen fertilization in 2021, 2022, and 2023.

Year	Straw	Nitrogen	SOC (g kg^-1^)	TN (g kg^-1^)	AP (mg kg^-1^)	AK (mg kg^-1^)	AN (mg kg^-1^)
2021	RT	—	12.39 ± 0.71	1.47 ± 0.05	13.66 ± 0.61	80.52 ± 2.79	59.68 ± 1.12
	DPR	—	13.24 ± 0.73	1.51 ± 0.07	15.19 ± 0.64	94.73 ± 2.73	65.8 ± 2.1
2022	RT	N0	11.15 ± 0.41a	1.31 ± 0.03d	13.07 ± 0.56a	73.27 ± 3.69b	54.9 ± 1.11d
		N1	11.32 ± 0.38a	1.37 ± 0.02c	13.32 ± 0.16a	78.88 ± 2.71a	60.87 ± 0.36c
		N2	11.67 ± 0.24a	1.43 ± 0.02b	13.58 ± 0.25a	80.49 ± 1.62a	63.85 ± 2.1b
		N3	11.92 ± 0.39a	1.49 ± 0.03a	13.84 ± 0.52a	82.91 ± 2.55a	67.64 ± 1.35a
	DPR	N0	13.37 ± 0.12c	1.52 ± 0.06c	14.43 ± 0.12c	89.98 ± 1.52c	67.08 ± 1.78c
		N1	13.87 ± 0.19bc	1.63 ± 0.04b	16.25 ± 0.35b	95.62 ± 2.10b	70.06 ± 1.69b
		N2	14.2 ± 0.23ab	1.74 ± 0.05a	17.06 ± 0.21ab	100.47 ± 1.95a	73.73 ± 2.43a
		N3	14.56 ± 0.17a	1.78 ± 0.06a	17.47 ± 0.29a	103.18 ± 1.85a	76.03 ± 3.02a
2023	RT	N0	11.19 ± 0.55b	1.25 ± 0.03d	12.80 ± 0.28b	71.8 ± 3.51b	49.41 ± 2.42d
		N1	11.38 ± 0.42a	1.33 ± 0.02c	13.75 ± 0.26a	80.38 ± 2.16a	63.08 ± 1.42c
		N2	11.74 ± 0.38a	1.42 ± 0.05b	13.96 ± 0.30a	84.19 ± 2.27a	68.96 ± 2.03b
		N3	11.98 ± 0.56a	1.52 ± 0.02a	14.22 ± 0.23a	87.33 ± 2.86a	74.41 ± 2.28a
	DPR	N0	13.5 ± 0.14c	1.57 ± 0.04c	14.71 ± 0.22c	91.39 ± 1.32c	67.85 ± 1.77c
		N1	14.42 ± 0.21b	1.71 ± 0.06b	16.74 ± 0.36b	98.12 ± 1.58b	78.11 ± 2.90b
		N2	15.24 ± 0.19a	1.84 ± 0.07a	18.25 ± 0.29a	103.45 ± 2.17a	84.52 ± 2.61a
		N3	15.58 ± 0.16a	1.89 ± 0.06a	18.96 ± 0.31a	105.16 ± 2.14a	88.95 ± 2.93a
Year (Y)			6.927*	5.790*	16.094**	6.819*	527.104**
Tillage (T)			359.589**	509.626**	156.007**	410.127**	397.441**
Nitrogen (N)			14.293**	68.440**	48.435**	41.052**	42.647**
Y×T			4.853*	18.582**	7.653**	0.035	182.875**
Y×N			0.818	2.545	1.278	0.721	25.813**
T×N			10.663**	3.011*	11.750**	26.239**	8.229**
Y×T×N			0.441	0.379	0.512	0.42	56.041**

SOC, Soil Organic Carbon; TN, Total Nitrogen; AP, Available Phosphorus; AK, Available Potassium; AN, Alkali-hydrolyzable Nitrogen. Values are presented as means ± standard errors. Different lowercase letters within the same column and year indicate significant differences at *p* < 0.05. Other values represent the F value of the analysis of variance. *indicates significance at *p* < 0.05; **indicates high significance at *p* < 0.01; ns indicates no significant difference at *p* > 0.05.

### 3.2 Soil organic carbon and total nitrogen

As shown in **Table 2**, the year had a significant effect on SOC and TN contents, while straw return and nitrogen application had highly significant effects. However, their three-way interaction (year × straw return × nitrogen) did not significantly affect either SOC or TN. Compared with the RT treatment, the DPR treatment significantly increased SOC content, with a total increase of 26.97% over the three-year period (*p*<0.05). SOC content in 2023 was 10.95% higher than in 2021. TN content under DPR in 2023 was significantly higher than that under RT, with an increase of 26.81% (*p*<0.05). TN content in 2023 was also 15.89% higher than in 2021 under the same treatment. Under the DPR treatment, SOC and TN contents decreased with the reduction in nitrogen application. In 2023, compared with N3, the N1 treatment significantly reduced SOC and TN contents by 8.04% and 9.52%, respectively (*p*<0.05). No significant differences were observed between the N2 and N3 treatments (*p*<0.05).

### 3.3 Soil labile carbon and nitrogen fractions

Soil labile carbon and nitrogen play important roles in enhancing soil fertility, stimulating microbial activity, and promoting nutrient cycling. As shown in **Table 3**, ANOVA results showed that year, straw return, and nitrogen application rate all had highly significant effects on soil easily oxidizable organic carbon (EOC), dissolved organic carbon (DOC), microbial biomass carbon (MBC), microbial biomass nitrogen (MBN), and mineral nitrogen (MN) contents. However, under the three-way interaction of these factors, only the MN content was significantly affected. Compared with the RT treatment, the DPR treatment significantly increased the contents of all measured carbon and nitrogen fractions (*p*<0.05). After three consecutive years of tillage, as observed in 2023, EOC increased by 37.16%, DOC by 27.18%, MBC and MBN by 27.18% and 43.96%, respectively, and MN by 52.55% (*p*<0.05). In 2023, compared with 2021 under DPR, EOC increased by 10.74%, DOC by 13.22%, MBC and MBN by 17.05% and 22.56%, respectively, and MN by 26.92% (*p*<0.05).

**Table 3:** Soil labile carbon and nitrogen fractions under different straw return treatments combined with nitrogen fertilization in 2021, 2022, and 2023.

Year	Straw	Nitrogen	EOC (g kg^-1^)	DOC (mg kg^-1^)	MBC (mg kg^-1^)	MBN (mg kg^-1^)	MN (mg kg^-1^)
2021	RT	N0	1.96 ± 0.05d	128.71 ± 6.12d	106.21 ± 3.97d	23.82 ± 0.57d	23.82 ± 0.57d
		N1	2.09 ± 0.09c	139.89 ± 5.69c	122.28 ± 5.70c	26.79 ± 0.51c	26.79 ± 0.51c
		N2	2.25 ± 0.07b	151.33 ± 3.17b	133.72 ± 3.17b	28.71 ± 1.01b	28.71 ± 1.01b
		N3	2.39 ± 0.06a	163.62 ± 5.34a	145.28 ± 5.83a	31.12 ± 0.64a	31.12 ± 0.64a
	DPR	N0	2.38 ± 0.09c	156.82 ± 5.07c	127.52 ± 6.84c	28.63 ± 1.12c	28.63 ± 1.12c
		N1	2.60 ± 0.09b	168.27 ± 5.49b	145.74 ± 5.54b	34.60 ± 0.65b	34.60 ± 0.65b
		N2	2.89 ± 0.11a	182.77 ± 6.23a	163.01 ± 2.91a	39.56 ± 0.86a	39.56 ± 0.86a
		N3	2.93 ± 0.13a	187.37 ± 4.01a	167.13 ± 5.38a	40.84 ± 1.57a	40.84 ± 1.57a
2022	RT	N0	1.86 ± 0.08d	125.84 ± 4.57d	110.69 ± 4.61d	21.98 ± 0.49d	21.98 ± 0.49d
		N1	2.11 ± 0.06c	142.69 ± 4.03c	126.19 ± 4.14c	28.34 ± 0.77c	28.34 ± 0.77c
		N2	2.30 ± 0.06b	156.73 ± 5.41b	140.13 ± 6.02b	31.23 ± 0.42b	31.23 ± 0.42b
		N3	2.45 ± 0.08a	169.53 ± 2.19a	150.59 ± 5.15a	34.65 ± 1.58a	34.65 ± 1.58a
	DPR	N0	2.49 ± 0.10c	160.79 ± 6.62c	130.17 ± 7.34c	27.76 ± 1.10c	27.76 ± 1.10c
		N1	2.76 ± 0.10b	177.69 ± 6.75b	155.94 ± 0.99b	38.32 ± 1.98b	38.32 ± 1.98b
		N2	3.08 ± 0.12a	194.54 ± 6.74a	179.76 ± 7.32a	47.33 ± 1.68a	47.33 ± 1.68a
		N3	3.15 ± 0.12a	202.97 ± 7.92a	182.48 ± 6.24a	48.99 ± 1.53a	48.99 ± 1.53a
2023	RT	N0	1.74 ± 0.10d	128.71 ± 6.12d	116.66 ± 0.80c	20.47 ± 0.68d	15.55 ± 0.20d
		N1	2.15 ± 0.08c	148.80 ± 3.01c	132.67 ± 4.93c	30.59 ± 0.66c	25.28 ± 0.54c
		N2	2.32 ± 0.07b	164.77 ± 5.53b	147.52 ± 4.86b	33.83 ± 1.03b	30.13 ± 0.23b
		N3	2.49 ± 0.05a	176.12 ± 5.45a	158.51 ± 5.45a	37.39 ± 0.91a	34.26 ± 0.71d
	DPR	N0	2.52 ± 0.07c	167.66 ± 6.99c	134.19 ± 5.00c	27.06 ± 1.10c	21.35 ± 0.39c
		N1	2.86 ± 0.11b	189.15 ± 4.55b	170.18 ± 9.52b	43.23 ± 1.70b	38.63 ± 0.53b
		N2	3.23 ± 0.11a	212.18 ± 7.52a	198.73 ± 8.08a	51.90 ± 2.66a	49.15 ± 1.33a
		N3	3.34 ± 0.09a	218.11 ± 4.95a	203.16 ± 7.93a	53.84 ± 2.30a	51.36 ± 1.43a
Year (Y)			7.66**	64.86**	41.15**	74.28**	71.51**
Tillage (T)			694.85**	1535.22**	1394.12**	1143.04**	4526.17**
Nitrogen (N)			124.76**	147.31**	118.49**	248.74**	1346.56**
Y×T			0.96	3.40*	7.441**	12.38**	32.10**
Y×N			0.02	1.00	0.42	2.24	66.61**
T×N			4.16*	4.01*	6.18*	11.65**	15.31**
Y×T×N			0.048	0.454	0.076	0.976	13.08**

EOC, Easily Oxidizable Organic Carbon; DOC, Dissolved Organic Carbon; MBC, Microbial Biomass Carbon; MBN, Microbial Biomass Nitrogen; MN, Mineral Nitrogen. Values are presented as means ± standard errors. Different lowercase letters within the same column and year indicate significant differences at *p* < 0.05. Other values represent the F value of the analysis of variance. *indicates significance at *p* < 0.05; **indicates high significance at *p* < 0.01; ns indicates no significant difference at *p* > 0.05.

Regardless of whether straw was returned, all fractions exhibited a decreasing trend with the reduction in nitrogen application rates. Under the DPR treatment, the contents of all fractions were significantly lower in N1 compared with N3, with EOC and DOC decreasing by 14.37% and 13.28%, MBC and MBN by 16.23% and 19.71%, respectively, and MN by 24.79% (*p*<0.05). At the N2 level, although the contents of these fractions were also reduced, the differences from N3 were not statistically significant (*p*<0.05). Under the RT treatment, all fractions showed significant decreases as nitrogen application rates declined.

### 3.4 Plant dry matter accumulation and nitrogen uptake by plants

As shown in **Table 4**, year, straw return, nitrogen application rate, and their two-way interactions all had highly significant effects on plant DMA and NUP. The three-way interaction of these factors had a significant effect on both DMA and NUP. In 2023, the DPR treatment significantly increased DMA and NUP by 23.22% and 74.38%, respectively, compared with the RT treatment (*p*<0.05). Additionally, after three years of straw return, DMA and NUP increased by 9.98% and 9.68%, respectively, compared to the first year (*p*<0.05). Under both straw return treatments, DMA and NUP generally declined with reduced nitrogen application. In 2023, under DPR, DMA was significantly lower in the N1 treatment than in N3 by 13.21% (*p*<0.05), while N2 showed a smaller, non-significant reduction. NUP in N1 and N2 was significantly lower than in N3, with reductions of 25.52% and 15.83%, respectively (*p*<0.05). Under the RT treatment, both DMA and NUP decreased significantly with the reduction in nitrogen application, NUP decreased by 40.39% and 22.43% in N1 and N2, respectively, compared with N3 (*p*<0.05).

**Table 4:** Plant dry matter accumulation and nitrogen uptake under different straw return treatments combined with nitrogen fertilization in 2021, 2022, and 2023.

Year	Straw	Nitrogen	DMA (g plant)	NUP (kg ha^-1^)
2021	RT	N0	175.59 ± 5.19d	44.03 ± 0.29d
		N1	228.27 ± 11.95c	73.67 ± 4.43c
		N2	262.51 ± 3.63b	111.91 ± 3.89b
		N3	283.51 ± 7.39a	137.68 ± 2.99a
	DPR	N0	256.40 ± 10.99c	94.35 ± 3.77d
		N1	321.14 ± 13.61b	140.38 ± 7.61c
		N2	345.253 ± 15.89ab	172.25 ± 6.15b
		N3	362.29 ± 10.33a	187.44 ± 6.54a
2022	RT	N0	191.81 ± 7.38d	43.43 ± 1.90d
		N1	259.51 ± 2.77c	75.30 ± 3.09c
		N2	297.13 ± 9.14b	101.14 ± 3.13b
		N3	348.00 ± 3.71a	136.97 ± 5.36a
	DPR	N0	273.67 ± 11.63c	98.14 ± 4.38d
		N1	326.18 ± 14.54b	128.48 ± 3.73c
		N2	357.36 ± 7.61a	152.24 ± 5.40b
		N3	377.69 ± 9.69a	169.45 ± 4.58a
2023	RT	N0	204.38 ± 6.70d	50.78 ± 2.91d
		N1	275.06 ± 13.53c	81.20 ± 1.26c
		N2	307.17 ± 8.54b	105.66 ± 2.31b
		N3	360.52 ± 9.69a	136.22 ± 9.00a
	DPR	N0	291.18 ± 9.63c	113.08 ± 5.05d
		N1	345.04 ± 7.98b	155.17 ± 8.40c
		N2	379.33 ± 13.85a	175.37 ± 7.77b
		N3	397.55 ± 15.28a	208.34 ± 5.59a
Year (Y)			87.81**	50.41**
Tillage (T)			631.66**	2237.06**
Nitrogen (N)			394.39**	957.91**
Y×T			18.26**	26.230**
Y×N			9.89**	3.87**
T×N			12.65**	5.38**
Y×T×N			2.95*	2.87*

DMA, Dry Matter Accumulation; NUP, Nitrogen Uptake By Plants. Values are presented as means ± standard errors. Different lowercase letters within the same column and year indicate significant differences at *p* < 0.05. Other values represent the F value of the analysis of variance. *indicates significance at *p* < 0.05; **indicates high significance at *p* < 0.01; ns indicates no significant difference at *p* > 0.05.

### 3.5 Yield and nitrogen efficiency

As shown in **Table 5**, year, straw return, nitrogen application rate, and their two-way interactions all had highly significant effects on maize yield, except for the three-way interaction, which had no significant effect. After three years of RT treatment, maize yield increased by 4.48% compared to the first year. Under the DPR treatment, the increase was 14.17%, indicating a more pronounced improvement with deep plowing with straw return. Maize yield decreased with reduced nitrogen application across all years. Under the DPR treatment, yield in the N1 treatment was significantly lower than in N3 by 5.08% in 2021, 4.13% in 2022, and 3.96% in 2023 (*p*<0.05). In contrast, yield under N2 was slightly lower than N3 in all years, but the differences were not statistically significant. These results suggest that the impact of nitrogen reduction on yield gradually weakened with continued straw return, highlighting the stabilizing effect of long-term straw incorporation under reduced nitrogen input.

**Table 5:** Maize yield and nitrogen fertilizer use efficiency under different straw return treatments combined with nitrogen fertilization in 2021, 2022, and 2023.

Year	Straw	Nitrogen	Yield (kg ha^-1^)	NUE (%)	PFPN (kg kg^-1^)
2021	RT	N0	10320.13 ± 254.86d	—	—
		N1	13078.28 ± 144.05c	14.12 ± 0.31c	62.28 ± 0.69a
		N2	13518.53 ± 83.15b	26.62 ± 1.04b	53.01 ± 2.09b
		N3	13903.33 ± 213.85a	31.22 ± 0.98a	46.34 ± 1.04c
	DPR	N0	10953.33 ± 695.14c		
		N1	14211.27 ± 20.00b	21.92 ± 0.93b	67.51 ± 3.66a
		N2	14610.00 ± 117.90ab	30.55 ± 1.15a	57.29 ± 1.96b
		N3	14966.67 ± 158.22a	31.03 ± 1.40a	49.56 ± 1.19c
2022	RT	N0	10227.95 ± 478.49d	—	—
		N1	13395.72 ± 124.48c	15.18 ± 0.60c	63.79 ± 1.85a
		N2	13902.17 ± 37.53b	22.83 ± 0.63b	54.75 ± 1.56b
		N3	14345.16 ± 132.09a	31.18 ± 1.02a	47.82 ± 0.44c
	DPR	N0	11283.33 ± 160.73c	—	—
		N1	15305.42 ± 193.37b	14.45 ± .16c	72.88 ± 0.92a
		N2	15927.82 ± 328.63a	21.22 ± 1.02b	62.46 ± 1.29b
		N3	15969.47 ± 387.32a	23.77 ± 1.04a	53.23 ± 1.29c
2023	RT	N0	10010.08 ± 500.89d	—	—
		N1	13746.67 ± 172.43c	14.48 ± 0.06c	65.46 ± 1.77a
		N2	14383.33 ± 145.72b	21.52 ± 0.87b	56.41 ± 1.33b
		N3	14990.00 ± 88.89a	28.48 ± 0.89a	49.98 ± 2.14c
	DPR	N0	11840.00 ± 655.97c	—	—
		N1	16490.00 ± 43.59b	20.05 ± 0.96c	78.52 ± 1.69a
		N2	17036.67 ± 98.66a	24.43 ± 0.54b	66.81 ± 1.28b
		N3	17170.16 ± 127.67a	31.75 ± 1.17a	57.23 ± 1.00c
Year (Y)			113.55**	112.74**	66.69**
Tillage (T)			594.29**	38.75**	259.75**
Nitrogen (N)			908.79**	932.55**	515.97**
Y×T			34.02**	92.33**	14.66**
Y×N			7.66**	14.49**	0.327
T×N			6.78**	44.59**	6.017**
Y×T×N			0.37	6.83**	0.496

NUE, Nitrogen Use Efficiency; PFPN, Partial Factor Productivity of Nitrogen. Values are mean ± standard error. Different lowercase letters within the same column in the same year indicate significant differences at *p* < 0.05. Other values represent the F value of the analysis of variance. *indicates significance at *p* < 0.05; **indicates high significance at *p* < 0.01; ns indicates no significant difference at *p* > 0.05.

Year, straw return, and nitrogen application rate all had highly significant effects on nitrogen use efficiency (NUE) and partial factor productivity of nitrogen (PFPN), but under the three-way interaction of these factors, only NUE was significantly affected. **Table 5** shows that, except for 2022, NUE was significantly higher under the DPR treatment than RT in 2021 and 2023, with increases of 16.01% and 18.24%, respectively (*p*<0.05). Under the DPR treatment, NUE declined with decreasing nitrogen input. For example, in 2023, NUE decreased significantly in N1 and N2 by 36.85% and 23.06%, respectively, compared with N3 (*p*<0.05). The DPR treatment significantly increased PFPN compared to the RT treatment, with increases of 7.87% in 2021, 13.36% in 2022, and 17.91% in 2023. In contrast, PFPN showed an increasing trend as nitrogen input decreased. In 2023 (*p*<0.05), under DPR, PFPN was significantly higher in N1 and N2 than in N3 by 37.20% and 16.74%, respectively (*p*<0.05). Similarly, under the RT treatment, PFPN increased significantly in N1 and N2 by 30.97% and 12.87%, respectively, compared to N3 (*p*<0.05). The increase in PFPN was more pronounced under nitrogen reduction treatments with straw return, indicating that nitrogen reduction under straw return conditions was more conducive to improving PFPN.

### 3.6 Prediction of optimal nitrogen application to maize under straw return conditions

**Figure 2:**
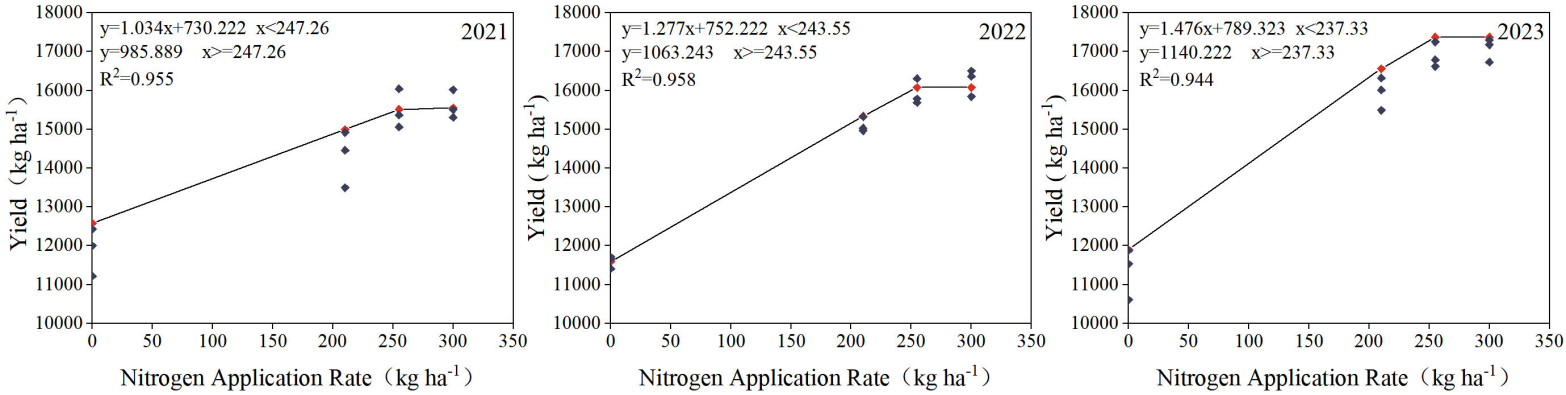
Effect of straw return combined with nitrogen fertilization on the optimal nitrogen application rate for maize.

As shown in **Figure 2**, maize yield exhibited a linear-plus-plateau relationship with nitrogen application rates. Under straw return conditions, the optimal nitrogen application rates for maize were 247.26 kg ha^-1^ in 2021, 243.55 kg ha^-1^ in 2022, and 237.3 kg ha^-1^ in 2023. After three years of straw return, the optimal nitrogen application rate for maize was 4.02% lower than in the first year and 20.89% lower than the conventional nitrogen application rate. These results suggest that straw return can effectively reduce the optimal nitrogen requirement for maize, and that the nitrogen-saving effect becomes more pronounced with increasing years of straw return.

### 3.7 Relationships between soil labile carbon and nitrogen, soil fertility, crop yield and nitrogen fertilizer utilization

**Figure 3:**
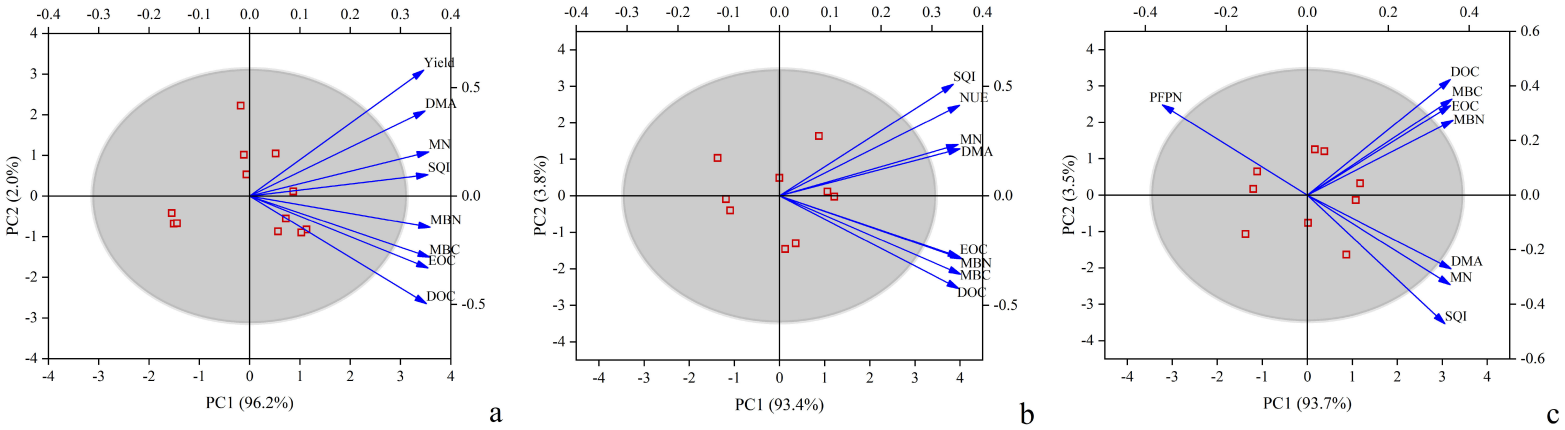
Principal component analysis (PCA) of multiple indicators under different straw return treatments. **(a)** PCA of crop yield; **(b)** PCA of nitrogen use efficiency (NUE); **(c)** PCA of partial factor productivity of nitrogen (PFPN). SQI stands for Soil Quality Index, DMA stands for Dry Matter Accumulation, EOC stands for Easily Oxidizable Carbon, DOC stands for Dissolved Organic Carbon, MBC stands for Microbial Biomass Carbon, MBN stands for Microbial Biomass Nitrogen, and MN stands for Mineral Nitrogen.

**Figure 4:**
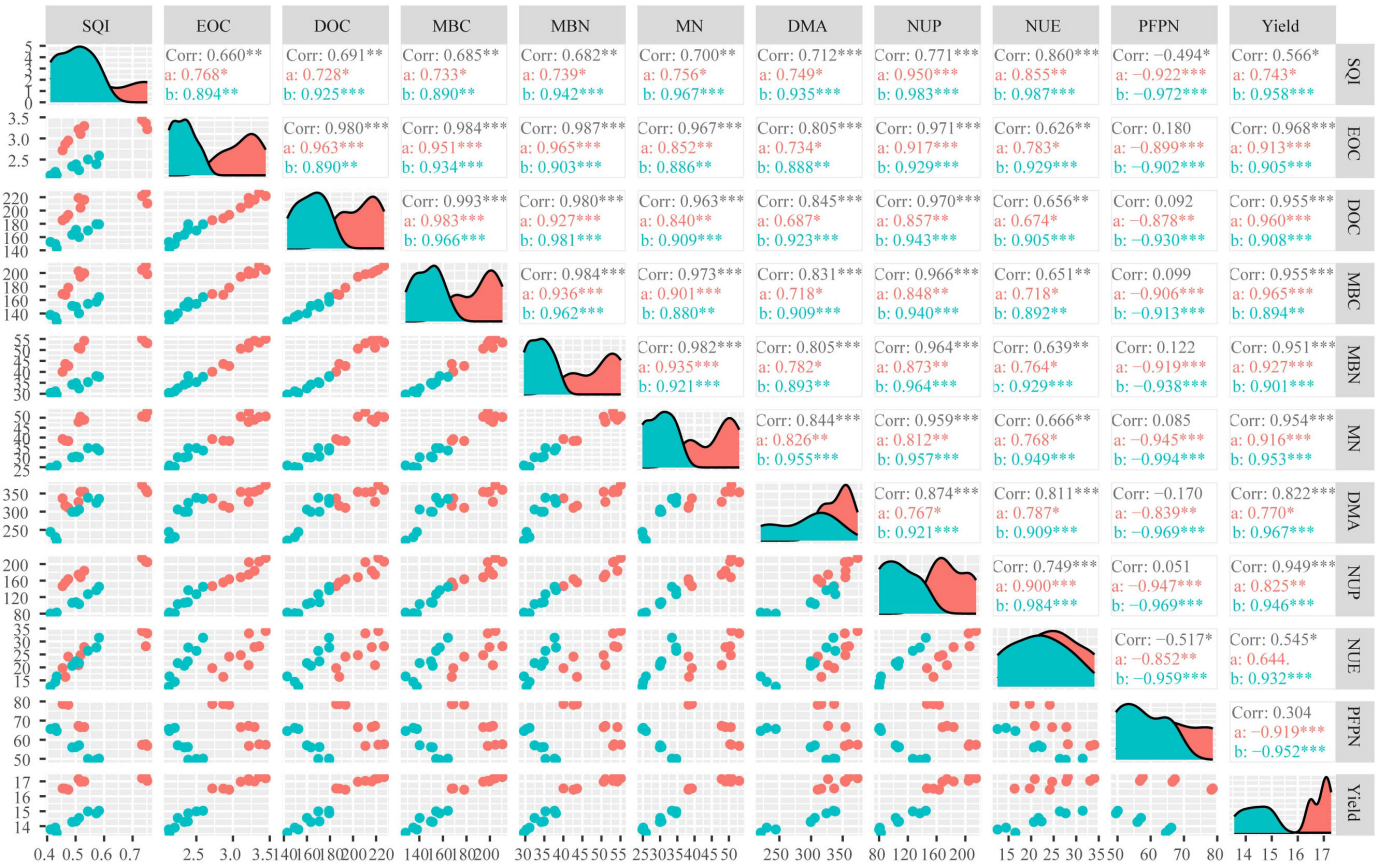
Correlation analysis among soil labile carbon and nitrogen fractions, maize yield, and nitrogen use efficiency.

Principal component analysis (PCA) was conducted based on soil labile carbon and nitrogen fractions, soil quality index (SQI), dry matter accumulation (DMA), yield, and nitrogen efficiency. The results showed that the cumulative contribution rates in **Figures 3a–c** were 98.2%, 97.2%, and 97.2%, respectively. The key contributing variables were mineral nitrogen (MN), SQI, and DMA, which played major roles in influencing yield and nitrogen efficiency. SQI, MN, and DMA were significantly positively correlated with yield and NUE but significantly negatively correlated with Partial Factor Productivity of Nitrogen (PFPN) (**Figure 4**). This suggests that good soil quality, high nitrogen availability, and sufficient plant DMA contribute to improving yield potential and nitrogen uptake. However, these factors may also limit the plant’s nitrogen utilization efficiency. Therefore, optimizing nitrogen fertilizer application, regulating soil organic matter content, and improving soil quality in agricultural management are beneficial for enhancing both crop productivity and resource use efficiency.

**Figure 5:**
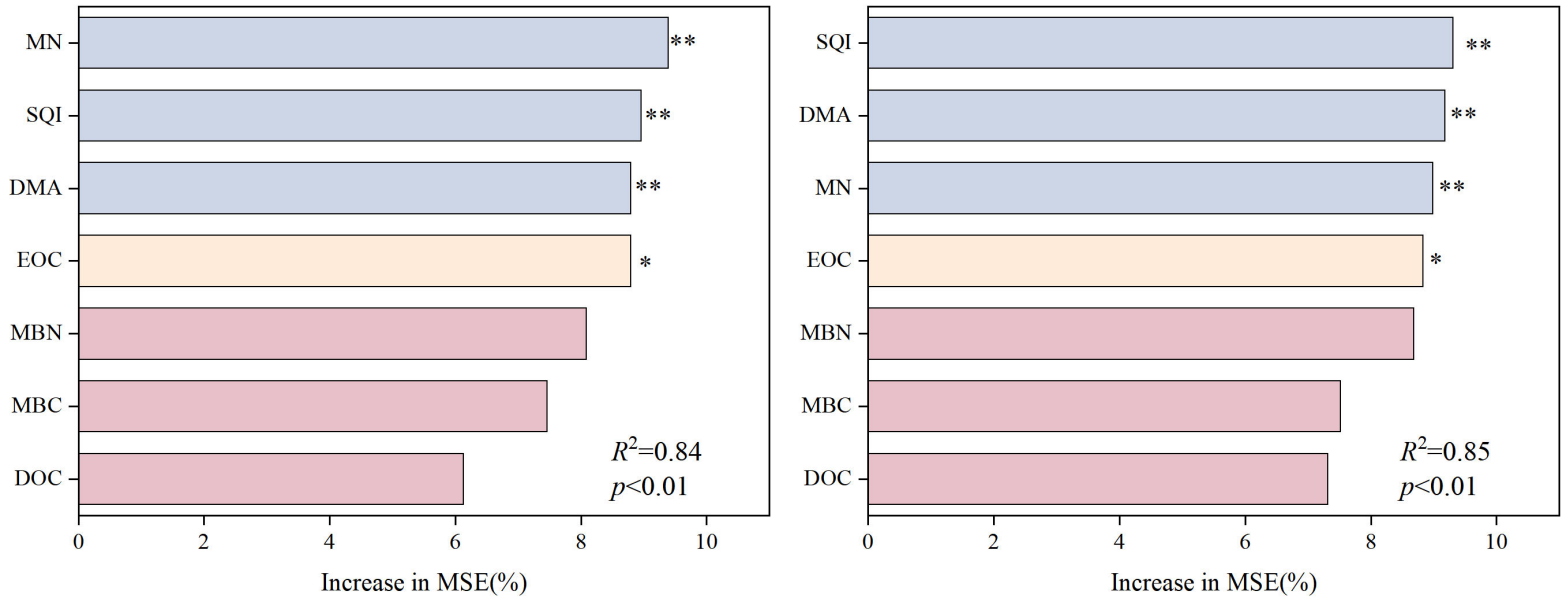
Random forest model analysis of nitrogen use efficiency (NUE) and partial factor productivity of nitrogen (PFPN). *indicates a significant effect at *p* < 0.05, **indicates a highly significant effect at *p* < 0.01, and ns indicates no significant effect at *p* > 0.05.

Further results from the random forest model (**Figure 5**) indicate that SQI, MN, and DMA are the most important factors influencing nitrogen efficiency (NUE and PFPN), with highly significant effects on both NUE and PFPN. These variables had highly significant effects on NUE and PFPN, with contribution rates of 9.39%, 8.96%, and 8.49% to NUE, and 9.31%, 9.18%, and 8.99% to PFPN, respectively. In contrast, other labile carbon and nitrogen fractions showed relatively weak direct effects on NUE and PFPN. These variables may influence NUE and PFPN indirectly by promoting microbial activity, accelerating the decomposition of organic matter and the release of MN, and improving soil fertility.

## 4 Discussion

### 4.1 Effects of straw return combined with nitrogen fertilization on soil fertility

In this experiment, the results showed that the DPR treatment significantly increased soil SOC and TN contents. Soil SOC and TN increased with higher nitrogen application rates, and there was no significant difference between the N2 and N3 treatments. These findings are consistent with those reported in previous studies (Zhao et al., 2018; Li et al., 2021). Under the combined application of straw return and nitrogen fertilization, sufficient nutrient inputs particularly nitrogen were provided, which accelerated the decomposition of straw by soil microorganisms and the release of nutrients into the soil. This process facilitated the conversion of more straw-derived carbon into SOC, thereby increasing SOC content (Chen et al., 2014b). Some studies have also reported that nitrogen application under straw return had no significant effect on SOC. This discrepancy may be attributed to the accelerated decomposition of microbial carbon in the soil following nitrogen application, which offset the additional carbon input from straw return. As a result, no significant change in SOC content was observed under nitrogen application combined with straw return (Chen et al., 2021). Returning straw to the field increases the nitrogen supply in the soil, provides essential nitrogen for microbial activity, and enhances the capacity of soil microorganisms to decompose straw, thereby releasing more nitrogen nutrients (Yang et al., 2023). Following nitrogen fertilizer application, the exogenous nitrogen input directly stimulates straw decomposition by soil microorganisms, resulting in a more pronounced improvement in soil nitrogen availability (Yang et al., 2017). Compared with RT, the DPR treatment significantly increased the contents of soil nitrogen (N), phosphorus (P), and potassium (K), with the highest levels of AN, AP, and AK observed under DPR combined with the N3 application rate. Since straw is rich in mineral nutrients, returning it to the field inevitably enhances nutrient accumulation in the surface soil layer, thereby playing an important role in improving the nutrient status of soil N, P, and K (Ghimire et al., 2017; Mukhametov et al., 2024). In addition, under the DPR treatment, the soil contents of N, P, and K at the N2 level were slightly lower than those at the N3 level, but the differences were not statistically significant. This indicates that, under reduced fertilizer input, straw return plays an important role in improving soil fertility. The decomposition of straw supplies the soil with abundant nutrients, including N, P, and K. Soil microorganisms, such as bacteria and fungi, play a crucial role in breaking down the organic matter from straw (Bhattacharyya et al., 2022). These microorganisms release microbial enzymes, such as cellulases and proteases, which break down complex organic compounds into plant-available forms, thus accelerating the mineralization and release of nutrients like N, P, and K (Ali et al., 2021). Meanwhile, the increase in organic matter enhances the activity of soil microorganisms and enzymes, thereby promoting nutrient release and improving nutrient availability (Chen et al., 2016). Microbial-driven processes, including enzyme activity and microbial metabolism, help improve soil health by increasing the concentration of readily available nutrients, which supports plant growth (Trivedi et al., 2021).

### 4.2 Effects of straw return combined with nitrogen fertilization on soil labile carbon and nitrogen fractions

Soil EOC and DOC are among the most labile carbon fractions in soil. Their concentrations can rapidly reflect changes in the soil organic carbon pool and are considered sensitive indicators for assessing early changes in soil carbon dynamics (Duval et al., 2018). The results of this study showed that soil EOC and DOC concentrations increased significantly under DPR combined with nitrogen fertilization, reaching the highest levels at the N3 application rate. Both EOC and DOC concentrations increased with the number of years of straw return, straw return increased carbon input to the soil and provided more carbon sources for microbial activity. During microbial decomposition, straw released greater amounts of dissolved organic matter, which is the main source of labile organic carbon in soil, thereby contributing to the increases in EOC and DOC concentrations (Ding et al., 2016). The results showed that soil MBC and MBN contents increased to varying degrees under straw return alone, nitrogen application alone, or their combination. MBC and MBN contents decreased with reduced nitrogen application and were highest under the N3 treatment. Under the DPR treatment, although MBC and MBN levels in N3 were higher than those in N2, the differences were not statistically significant. Previous studies have also reported that soil MBC and MBN contents were significantly lower under straw return combined with nitrogen fertilization than under straw return alone (Cao et al., 2022). One possible explanation for the differing results is the variation in nitrogen demand among different crops. The contents of soil MBC and MBN are influenced by nitrogen availability, which is subject to a biological threshold. When nitrogen availability exceeds this threshold, microbial activity may be inhibited. In some previous studies, the nitrogen application rates exceeded the recommended levels for the target crops, resulting in nitrogen availability surpassing the thresholds for MBC and MBN and consequently leading to a reduction in their contents (Xie et al., 2021; Rosinger et al., 2022). Secondly, the use of long-term excessive application of nitrogen fertilizer alone may lead to an imbalance of other essential nutrients in the soil. Under such nutrient-deficient conditions, increasing nitrogen availability alone is insufficient to enhance soil microbial biomass or activity (Dai et al., 2018). In this study, the nitrogen application rate at the N3 level may have approached, but did not exceed, the critical threshold throughout the crop growing season. Moreover, long-term high nitrogen input can aggravate the imbalance of soil mineral nutrients and exert negative effects on soil microbial communities (Ma et al., 2023). Nitrogen uptake and utilization by plants primarily occur in the form of inorganic nitrogen. The level of soil inorganic nitrogen is a key indicator for assessing soil nitrogen availability (Moreau et al., 2019). The results of this experiment showed that the MN content in the 0–100 cm soil layer was significantly increased under DPR combined with nitrogen fertilization, reaching the highest level at the N3 treatment. This was attributed to the additional nitrogen source provided by DPR, which created a decomposition zone in the subsoil, accelerated straw decomposition, and consequently promoted the accumulation of MN in the soil (Huang et al., 2021), At the same time straw return may have altered soil microbial, nitrification and denitrification processes, greatly promoting microbial growth and inorganic nitrogen fixation (Wu et al., 2023), Under the conditions of this experiment, straw return and nitrogen application were the primary driving factors influencing the accumulation of soil MN. However, MN accumulation is also affected by multiple factors, such as the soil C/N ratio, moisture, temperature, and pH (Zhang et al., 2022), The extent and significance of these influences require further investigation in future studies. Although this experiment did not involve observations of changes in microbial communities, previous studies have shown that soil microorganisms alter the soil carbon and nitrogen stocks through decomposition and formation of soil organic matter (Fan et al., 2021). Straw return can significantly affect the abundance of dominant microbial phyla in the soil, such as Proteobacteria, Acidobacteriota, and Bacteroidota, and these microbial changes are closely associated with soil labile carbon components, such as DOC and MBC (Wu et al., 2021a; Wang et al., 2025). Furthermore, increased nitrogen input can enhance the relative abundance of Proteobacteria and Bacteroidota, thereby accelerating soil nitrogen cycling and improving carbon use efficiency (Wang et al., 2023). In addition, Mortierellomycota, a fungal group commonly associated with straw decomposition, has been identified as a key contributor to the turnover of organic polymers such as chitin and cellulose, further enriching the soil carbon and nitrogen pools (Ozimek and Hanaka, 2020; Wolińska et al., 2022). In summary, the combined application of straw return and nitrogen fertilization not only contributes to enhancing the soil carbon and nitrogen pools, but also regulates the biogeochemical cycling of soil nutrients by stimulating the response of microbial communities.

### 4.3 Effects of straw return combined with nitrogen fertilization on maize yield and nitrogen efficiency

The results of this study showed that the DPR treatment significantly increased maize yield. Yield decreased with the reduction in nitrogen application, with the highest yield observed under the N3 treatment. Although the yield under N2 was slightly lower than that under N3, the difference was not statistically significant. Under the DPR treatment, the model-predicted optimal nitrogen application rate could be reduced to 237.3 kg ha^-1^ while maintaining a high yield level. In the semi-arid regions of the Loess Plateau, under straw return conditions, the optimal nitrogen fertilizer application rate for nitrogen efficiency and yield is generally between 200 kg ha^-1^ (Xie et al., 2022), while in the black soil region of Northeast China, the optimal nitrogen rate under continuous straw return can be reduced to 180–220 kg ha^-1^ (Song et al., 2024). In this study, the optimal nitrogen rate was relatively higher, which may be due to high soil fertility, favorable water conditions, and long-term straw return that can enhance the soil nitrogen supply capacity and accelerate nitrogen mineralization rate, thereby allowing a significant reduction in nitrogen input without affecting yield and nitrogen efficiency; otherwise, a higher nitrogen rate is needed to maintain yield (Wang et al., 2024). Previous studies have also reported that crop yield may decrease under straw return combined with high nitrogen application. This may be attributed to excessive nitrogen input promoting overly dense plant populations, which can hinder ear development, reduce the number of kernels per ear, lower grain weight, and ultimately result in decreased final grain yield (Shan et al., 2021). Secondly, excessive nitrogen fertilization may cause high nitrogen stress, which suppresses crop growth and development. This can lead to excessive vegetative growth and delayed maturity, resulting in a reduced number of effective ears and lower grain filling. There are inflection points and threshold levels in the crop’s NUE, when nitrogen application exceeds a certain level, grain yield tends to plateau or even decline (Jiang et al., 2024). The results of this study showed that the DPR treatment significantly increased NUE and PFPN. However, with decreasing nitrogen application rates, NUE tended to decline, while PFPN exhibited an increasing trend. Both NUE and PFPN reflect nutrient uptake efficiency. The observed difference in results may be attributed to the fact that PFPN reflects the yield obtained per unit of nitrogen input, while NUE reflects the crop’s response to exogenous nitrogen uptake (Mahboob et al., 2023). In this study, under nitrogen reduction conditions, although crop yield decreased, it remained stable within a certain range of nitrogen reduction, thereby improving the input-output efficiency of nitrogen, and resulting in an increase in PFPN with decreasing nitrogen application. However, regarding NUE, with increasing nitrogen application rates, plant nitrogen uptake showed a significant increasing trend, indicating that the soil’s inherent nitrogen-supplying capacity was limited, and the crop had not yet reached a saturation state of nitrogen uptake (Xu et al., 2023; Zhang et al., 2024a). Nitrogen application significantly improved nitrogen uptake efficiency, leading to higher NUE under high-nitrogen conditions (Zhang et al., 2020). In addition, consecutive years of straw return combined with nitrogen fertilization improved the nitrogen transformation process in the soil and enhanced nutrient uptake capacity in the plant rhizosphere, thereby promoting nitrogen uptake and contributing to the increase in NUE (Xu et al., 2024). However, other studies have shown that with increasing nitrogen application rates, NUE tends to decline, while PFPN remains stable or even increases. The discrepancy in results may be due to excessive nitrogen inputs at higher application rates, which can lead to nitrogen losses or over-uptake by crops, thereby reducing NUE (Wu et al., 2021b; Shi et al., 2023); Secondly, differences in nitrogen fertilizer use efficiency can also be attributed to factors such as soil type, organic nutrient content, crop type, and the number of years of fertilization (Chen et al., 2022).

### 4.4 Soil labile carbon and nitrogen, soil fertility in relation to yield and nitrogen efficiency

Based on principal component analysis and random forest model results, dry matter accumulation (DMA), mineral nitrogen (MN), and the soil quality index (SQI) were identified as the key factors influencing nitrogen efficiency. DMA reflects the overall capacity of the crop to absorb and assimilate nutrients and is closely related to nitrogen uptake and conversion efficiency, thereby directly contributing to yield formation and nitrogen efficiency (Li et al., 2025). MN, as the main nitrogen source readily available to plants, plays a central role in regulating nitrogen nutrition and metabolism, directly determining nitrogen efficiency (Li et al., 2024a). In this experiment, SQI, which integrates indicators such as SOC, TN, and available N, P, and K, serves as a comprehensive measure of soil nutrient supply capacity and fertility status. A higher SQI implies better soil structure and enhanced synchrony between nutrient availability and crop demand, which contributes to improving nitrogen efficiency (Liu et al., 2025).

Although labile carbon and nitrogen components (such as EOC, DOC, MBC, and MBN) are indirect factors affecting nitrogen efficiency, they contributed indirectly to nitrogen efficiency by stimulating microbial activity and enhancing nutrient turnover (Duo et al., 2025). These components promoted the transformation and mobilization of MN in soil, thus improving nutrient availability (Quan et al., 2020). SOC, as a core component of SQI, also enhanced soil buffering capacity and microbial habitat stability, playing a supportive role in maintaining soil fertility and sustaining nitrogen efficiency (Zhang et al., 2025). The combined application of straw return and nitrogen fertilization significantly increased SOC and labile carbon and nitrogen pools, thereby improving both MN supply and SQI, ultimately contributing to the coordinated enhancement of crop yield and nitrogen efficiency (Li et al., 2024b). These findings provide theoretical support for optimizing nitrogen input under straw return practices to achieve efficient and sustainable agricultural production.

### 4.5 Limitations and future perspectives

This study provides practical evidence for optimizing nitrogen management under long-term straw return. The results suggest that under continuous straw incorporation, moderate nitrogen reduction can sustain high soil fertility, maintain crop yield, and enhance nitrogen efficiency, reflecting both productivity and sustainability. From a policy perspective, integrated practices of straw return and nitrogen optimization are recommended in areas with improved soil quality to promote sustainable agriculture. However, the study is limited by its spatial and temporal scope. The SQI used did not include microbial indicators, and nitrogen losses to the environment were not assessed. Future research should extend across diverse regions and timeframes, incorporating microbial functions and environmental nitrogen losses to better elucidate the mechanisms driving soil quality and nitrogen efficiency.

## 5 Conclusion

Successive years of straw return combined with nitrogen fertilization enhanced soil nutrient supply, increased labile carbon and nitrogen contents, and promoted plant DMA, thereby improving crop yield and nitrogen efficiency. Soil fertility level, MN, and plant DMA had significant effects on maize yield and nitrogen efficiency. In contrast, other components influenced yield and nitrogen efficiency indirectly by enhancing microbial activity and promoting the release of MN. Under the DPR treatment, no significant differences were observed in the contents of various soil and plant components or in maize yield between the 15% reduced nitrogen application and the normal nitrogen application. After three consecutive years of straw return, the optimal nitrogen application rate for maize was significantly lower than the conventional rate. The linear-plus-plateau prediction models and principal component analysis (PCA) plots. model predicted that the optimal nitrogen rate could be reduced to 237.3 kg ha^-1^. By supplying organic carbon and nitrogen sources, straw return promotes microbial activity, accelerates the mineralization of organic nitrogen, and provides available nitrogen for plant uptake. At the same time, it improves soil fertility and enhances the soil’s nitrogen-supplying capacity, thereby partially substituting for synthetic nitrogen fertilizer and reducing dependence on external nitrogen inputs. Considering the improvement of soil organic matter, the enhancement of soil fertility, the promotion of agricultural ecosystem sustainability, and the advancement of green agriculture, DPR combined with a nitrogen application rate of 237.3 kg ha^-1^ can not only improve soil quality and crop productivity more effectively, but also reduce environmental risks. This practice can be recommended as an optimal field management strategy for the Tumochuan Plain region.

## Data Availability

The raw data supporting the conclusions of this article will be made available by the authors, without undue reservation.
